# On the Means of Supplying the Poor More Effectually with Medical Assistance in the Country Parts of Ireland

**Published:** 1806-03

**Authors:** 


### On the Means of supplying the Poor more effectually with Medical Assistance in the Country Parts of Ireland.

It is now several years since the Legislature of this country, taking into consideration the distressed and helpless less state of the poor in the provinces, who from accident or disease were deprived at the same time of health and of the means of subsistence, voted several considerable funds for the erection and endowment of hospitals, to be provided in the most central and populous town of each county.—That these establishments should be supplied with sufficient medical assistance, it was enacted that a surgeon, duly qualified, should be appointed to each, by the governors of the county; and in appointing a surgeon instead of a physician, it was wisely considered that the medical education of the former would enable him to afford relief in most diseases to which the poor are subject; whereas the latter would be incompetent to perform surgical operations when necessary.

Each hospital is amply supplied with all manner of surgical instruments, and apparatus, and with whatever medicines the surgeon may require. The buildings are in general large and commodious; beside a spacious vestibule, a large saloon for a surgery or pharmacy, and suitable apartments for a nurse, porter, &c. there are accommodations for about fifty patients.

The regular funds for the support of these establishments are, as I am informed, about three hundred pounds a year; beside, the casual subscriptions from gentlemen of the county, charity balls, &c. &c. may possibly amount, upon a moderate computation, to about one hundred and fifty pounds more, with a very small share of exertion on the part of the governors or surgeon. Upon these funds twenty-five patients in acute diseases or severe injuries might be amply provided for, and the expences of house repairs, servants wages, medicines, &c. defrayed.

One half of the wards would still remain untenanted; and as there are many poor persons unable to pay for medical assistance, whose circumstances would yet enable them very well to maintain themselves, with such patients the remaining wards might be filled.

Very different, however, is the administration of the county hospitals in Ireland, or at least of the generality of them. One third of the established fund goes by way of sinecure salary to some surgeon or obstetrical practitioner, who usually having the largest share of practice in the neighbourhood, gives himself as little concern as possible about the relief of the poor, generally deputing the hospital practice to an apprentice, who has had perhaps six, twelve, or eighteen months experience in his profession. The number of patients accommodated in the house house seldom exceeds six or seven with trivial ailments, the more considerable injuries not frequently occurring in the country; and the doctor declaring that the intention of the institution is confined to surgical patients, excuses himself from receiving these acute cases, which are almost exclusively those that among the poor require medical assistance. The far greater number of wards being therefore shut up, may be hired out to lodgers by the surgeon, as I am informed has actually been done in one or two instances. How the remainder of the funds of the county hospitals are disposed of, I do not pretend to say; the surgeon is, I believe, the principal administrator, and his accounts are occasionally laid before the patrons by whom he has been appointed. Yet these gentlemen do not think that their merit and labours in the service of the poor are sufficiently remunerated; or that the revenues of their country are sufficiently exhausted by the numerous sinecure appointments, and gratuitous pensions that are already squandered on men who have as few pretensions as themselves. Like the unprincipled crew of a sinking vessel, they desire that they also should partake of the spoils, and have actually resolved to petition parliament for *another third* of the fixed funds of the charity. While the sick of an extensive parish in England are not only supplied with medical attendance, but with medicine also for ten pounds a year, though they are widely dispersed through the country, the few patients that seek relief at an Irish county hospital are collected under the eye of the surgeon. In the cities of Ireland the case is far different. Here, abundant hospitals are erected and endowed for the relief of the poor, and medical practitioners are emulous of the honour of being elected to administer their elymosynary aid.

To effect the object then, of " more effectual medical assistance to the poor in the provincial parts of Ireland," I would humbly propose that each subscriber, to a certain amount, be qualified to vote for the election of a surgeon to a county hospital; and it is probable that the majority of a *numerous* body would fix on a person of probity and high professional reputation. The salaries hitherto paid I would propose to withdraw, and I make no doubt that very many medical men of the highest respectability in the several counties would eagerly come forward to contend for the appointment, and devote to it their *real* services, and that the number of subscriptions would be considerably augmented from the institution becoming really useful, ful, from the ambition of having a vote in the election of a surgeon, and from the probability of obtaining accommodation in the house for the patients they might recommend. The plan is not novel, I believe it is the plan on which the generality of county hospitals in England, as well as the hospitals in the Irish cities, are conducted.

ANTIPECULATOR.

*Kilkenny, January* 13, 1806.

